# Synergistic effects of CTAB and low salinity brines on asphaltene behavior and emulsion stability in clay rich sandstone reservoirs

**DOI:** 10.1038/s41598-025-23167-9

**Published:** 2025-11-12

**Authors:** Zakarya Ahmed, Ali B. M. Ali, Omar J. AlKhatib, Ibrahim Mahariq, Karim Kriaa, Vahed Saif

**Affiliations:** 1 College of Engineering, Imam Mohammad Ibn Saud Islamic University (IMSIU), Riyadh, 11432 Saudi Arabia; 2 Advanced Technical College, University of Warith Al-Anbiyaa, Karbala, Iraq; 3 Architectural Engineering Department College of Engineering, UAE University, Al Ain, United Arab Emirates; 4 Department of Medical Research, China Medical University Hospital, China Medical University, Taichung, Taiwan; 5 University College, Korea University, Seoul, 02481 South Korea; 6 Faculty of Engineering and Technology, The Technological University of Tajikistan, Dushanbe, Tajikistan

**Keywords:** Heavy oil, Clay particles, Asphaltene, Smart water, Emulsion, Zeta potential, Energy science and technology, Engineering

## Abstract

Improving oil recovery in sandstone reservoirs with higher concentrations of clay particles (clay-rich) presents a persistent challenge, especially in heavy oil extraction. Although low-salinity water flooding has been investigated for sandstone reservoirs, the synergistic effects of heavy oil molecular composition, cationic surfactants (e.g., cetyltrimethylammonium bromide, CTAB), clay particles, and ion-tuned brines on emulsion stability and oil recovery remain poorly understood. This study investigated the molecular behavior of asphaltene under the synergistic effects of CTAB and low salinity water flooding in clay-rich systems. Advanced experimental techniques, including interfacial tension (IFT) measurements, viscosity analysis, and zeta potential assessment, revealed that sulfate-enriched seawater (SW5d.3SO4) in the presence of clay and CTAB hindered asphaltene migration. However, cation-enriched seawater (SW5d.3Mg) promoted asphaltene migration, increasing IFT by ~ 18 mN/m to 48.23 mN/m and decreasing viscosity by approximately 351.3 cP to 249.5 cP. ATR (Attenuated total reflection)-FTIR (Fourier transform infrared spectroscopy) analysis demonstrated that sulfate-rich brines preferentially mobilized less-polar components, whereas cation-rich brines reduced the polar content of the oil phase. Additionally, (SW5d.3Mg) increased the asphaltene onset point precipitation (AOP) by 11% and reduced asphaltene concentration by ~ 5%, enhancing flow assurance. These findings provide critical insights into emulsion stabilization mechanisms and fluid-rock interactions, offering a sustainable strategy to optimize low-salinity water flooding with CTAB for enhanced heavy oil recovery in sandstone reservoirs.

## Introduction

Although worldwide initiatives have sought to reduce reliance on petroleum by diversifying energy supplies, hydrocarbon resources, particularly oil and gas, continue to dominate global energy sources^1–3^. As conventional oil reserves diminish due to prolonged extraction, innovative techniques have been developed to mitigate declining reservoir pressure^2,4–7^. These advanced approaches, which modify the physicochemical characteristics of reservoirs to improve hydrocarbon extraction, are known as Enhanced Oil Recovery (EOR) technique^2,4,8,9^. Chemical methods utilized in the EOR process include surfactant and ion-tuned water (smart water or low salinity water), and the simultaneous use of these chemicals. Among these techniques, smart water flooding is widely utilized in EOR^1,10,11^.

Recently, significant research efforts have focused on refining the ionic composition of injection brine, a technique termed smart water flooding, to maximize light oil recovery from sandstone reservoirs^12–14^. This advanced methodology employs strategic modification of brine chemistry through controlled adjustment of key ionic species - particularly sulfate (SO₄²⁻), calcium (Ca²⁺), and magnesium (Mg²⁺) concentrations - to optimize interfacial interactions and enhance crude oil mobilization within the sandstone reservoir^10^. Nevertheless, alterations of the salinity may destabilize the equilibrium of the oil-water-rock system, particularly in heavy oil reservoirs^15^. Such unintended interactions can significantly compromise the efficiency of enhanced oil recovery operations.

Recent research has highlighted the critical influence of clay minerals in sandstone reservoirs on low-salinity water flooding performance in heavy oil systems^16^. This significance stems from the complex interfacial phenomena that occur when heavy polar compounds in viscous crude oils interact dynamically with both aqueous phases and clay surfaces, leading to wettability alterations and oil recovery behaviors^17,18^. The substantial role of clay minerals in such reservoir systems has been fundamentally explained through the Multi-Ion Exchange (MIE) mechanism, originally developed by Lager et al.^16,19^. Current research proposed that clay minerals - particularly kaolinite groups with elevated cation exchange capacity (CEC) - serve as pivotal agents in smart water flooding processes^20,21^. Consequently, recent scientific inquiry has increasingly focused on elucidating the complex interfacial dynamics between heavy hydrocarbons, ion-tuned water, and reservoir’s minerals during water flooding operations.

While smart water flooding demonstrates considerable potential for improved hydrocarbon recovery, its application in sandstone reservoirs can induce complex fluid behavior, most notably through emulsion formation^22^. These interfacial phenomena exhibit a dualistic impact on recovery processes: (1) enhancing microscopic displacement efficiency through improved mobility control, while (2) potentially impairing sweep efficiency due to increased fluid viscosity and pore throat blockage, especially water-in-oil (W/O) emulsions^23,24^. These emulsion-induced phenomena create substantial operational and economic challenges, with particularly severe consequences in surface processing facilities^24^. Consequently, the development of robust mitigation strategies to control water-in-oil emulsion formation and optimize production system stability is critical for sandstone reservoirs under the smart water flooding process.

A critical challenge in smart water flooding is the amplification of emulsion generation, driven by complex interfacial interactions between injected brines, clay minerals, and heavy constituents of crude oil. Sandstone reservoirs typically contain reactive clay species—notably kaolinite, illite, and montmorillonite—which actively participate in interfacial phenomena that significantly influence their interactions with heavy components of oil, including asphaltene molecules^22^. Asphaltenes possess high molecular weight polycyclic aromatic structures with polar heteroatoms (N, O, S), enabling them to accumulate at oil-water interfaces, reduce interfacial tension, and form rigid films through irreversible adsorption^25^. For example, Valdes et al. investigated the influence of asphaltene compounds on emulsion generation in crude oil systems. Their research demonstrated that modifying the resin-to-asphaltene (R/A) ratio significantly improved the stability of emulsion^26^.

Liang et al. demonstrated that the concentration of polar components of oil, including asphaltenes and NSO (nitrogen, sulfur, and oxygen) compounds, had considerable capability in modifying oil-water interfacial properties, influencing emulsion stability^27^. Also, Joonaki et al. showed that high salt concentrations in aqueous phases promote the formation of larger, poly-aromatic asphaltene aggregates, affecting their interfacial behavior^28^. Elkhatib et al. studied how asphaltene molecular structure influences emulsion behavior, finding that aliphatic chains tend to form stronger aggregates^29^. The influence of oil components’ structure and concentration, particularly asphaltenes, on emulsion behavior is crucial to study.

Recent studies have also explored the impact of ion-tuned water and mineral particles on emulsion behavior, revealing their role in altering interfacial properties, stability, and phase interactions. Balavi et al. investigated the synergistic effects of cations and calcite on asphaltene adsorption and emulsion stability. Their study demonstrated that high-salinity conditions enhance asphaltene adsorption onto rock surfaces, significantly altering emulsion stability^30^. Cai et al. demonstrated that kaolinite nanotubes effectively stabilize Pickering emulsions, significantly improving their stability^31^.Besides, Mahdavi et al. revealed that the combined effect of salinity, clay particles, and polar crude oil components (e.g., asphaltenes) led to the formation of highly stable emulsions^22^. Zhou et al. investigated the efficacy of silica nanoparticles in stabilizing W/O emulsions. Their results highlighted that the surface chemistry and interfacial activity of particles significantly improve emulsion stability^32^.

Surfactants such as cetyltrimethylammonium bromide (CTAB), as a cationic surfactant, have been widely investigated for their ability to mitigate emulsion-related challenges in oil recovery, modify wettability, and lower interfacial tension (IFT)^1,33^. CTAB’s amphiphilic nature—featuring a positively charged hydrophilic head and a hydrophobic tail—facilitates interactions with both polar and less-polar components in reservoirs, thereby affecting emulsion stability^1,34^. Many studies have explored the impact of CTAB on emulsion formation and stabilization.

To investigate the interaction of surfactant, fresh oil fractions and particles, researchers employed a multidisciplinary approach, integrating experimental techniques to analyze interfacial behavior and emulsion stability. In this way, key analytical and experimental methods include Fourier-transform infrared spectroscopy (FTIR) for chemical characterization, interfacial tension (IFT) analysis, and zeta potential evaluation, can be conducted^35–39^. For example, Seng et al. revealed that saturated components in heavy oils promote the formation of more uniform and smaller droplets in the emulsion phase when interacting with surfactants^40^. In a related study, Koreh et al. investigated the interfacial behavior of different surfactants and their effects on oil-water interface dynamics. Their results demonstrated that surfactant selection critically influences emulsion stability^33^.

The research conducted by Divandari et al. examined the influence of nanoparticles (NPs) low salinity water and surfactant solutions. Their findings indicate that while a surfactant solution can alter the interfacial tension, mixing brine, surfactant, and NPs have a more pronounced effect on IFT^41^. Hou et al. showed that mixing of surfactants increased their adsorption on mineral surfaces, increasing rock water-wetness^42^. In a follow-up study, they found that gemini surfactants significantly enhance oil recovery by declining asphaltene accumulation at the mineral surface and interfacial tension^43^. A comprehensive study can help clarify the simultaneous effect of surfactant, oil fractions, low salinity water, and clay within the fluid-fluid interface.

This study investigates the emulsion stability during the examination of the synergistic interactions between the cationic surfactant cetyltrimethylammonium bromide (CTAB), clay particles, and key seawater ions (Ca²⁺, Mg²⁺, and SO₄²⁻) with fresh oil hydrocarbons, with a particular focus on the molecular structure of asphaltene fractions. An innovative combination of molecular-level analysis and carefully controlled sample preparation was employed to replicate reservoir-relevant conditions. The aged oil phase, separated from long-term aged emulsions, was characterized using interfacial tension (IFT) measurements, zeta potential analysis, asphaltene onset precipitation (AOP), and IP-143 tests to evaluate interfacial and compositional changes. Furthermore, ATR–FTIR spectroscopy was applied to elucidate the molecular structure and functional groups of the aged oils’ asphaltenes. The relationship between asphaltene molecular structure and emulsion stability was quantified by monitoring phase fraction distributions.

## Materials and methods

### Materials

#### Crude oil

The characteristics of the crude oil employed in this experimental investigation are presented in Table 1. Notably, previous studies have indicated that this crude oil exhibits properties similar to those of heavy oil^22,44^. The Colloidal Instability Index (CII) was measured to evaluate heavy oil stability using Eq. 1. A CII value exceeding 0.9 suggests that the crude oil contains unstable asphaltenes^45^.

1 $$CII = {\text{ }}\left( {saturation + {\text{ }}asphaltene} \right){\text{ }}/{\text{ }}\left( {re\sin + {\text{ }}aromatic} \right)$$

**Table 1:** Properties of the fresh oil utilized in this experimental study.

Fresh oil properties (unit)	Result
Density@25°C (g/cm^3^)	0.92
Acid number (mg KOH/g oil)	0.66
Asphaltene (%mass)	12.83
Resin (%mass)	15.36
Saturates	35.29
Aromatic	36.52
CII	0.93

#### Smart water

To assess the synergistic effects of various ions on the performance of CTAB and clay particles, this study utilized synthetic ion-tuned brines, along with distilled water. Synthetic seawater, based on Arabian Sea composition, was used as the base for preparing smart water solutions. Salts used in brine preparation were of analytical grade (> 99% purity) and were purchased from Merck (Darmstadt, Germany). To examine the effect of salinity reduction and individual ion enhancement:

Initially, the seawater (SW) was first diluted to 50% strength by mixing equal volumes of SW and deionized water, producing a diluted solution labeled SW5d (i.e., seawater diluted half times by weight with distilled water). This dilution was performed to prevent salt precipitation when the ion concentration increased, the seawater solution was initially diluted to 50% strength by gradually adding deionized water.

To investigate the individual effect, calcium, and magnesium ions, three ion-enriched brines were prepared by adding three times of specific salts to the diluted base (SW5d). In (SW5d.3SO4), the concentration of sodium sulfate (Na₂SO₄) was increased threefold compared to the original concentration in SW, keeping other components constant. In (SW5d.3Ca) and (SW5d.3Mg), calcium chloride (CaCl₂) and Magnesium chloride hexahydrate (MgCl₂·6 H₂O) concentrations were increased threefold over the original SW concentration, respectively.

Each brine solution was prepared by sequentially dissolving the appropriate salts in diluted SW5d under constant magnetic stirring (200 rpm) at room temperature (25 °C). After preparation, all solutions were allowed to stabilize for at least 30 min to ensure complete dissolution and achieve ionic homogeneity. Subsequently, the cationic surfactant CTAB (molecular weight: 348.47 g/mol; critical micelle concentration (CMC): 0.386 g/L) and clay particles were then added to each brine at fixed concentrations—0.386 g/L for CTAB (equal to its CMC) and 1.5 g for clay particles—to maintain consistent surfactant activity across all formulations. The final Total Dissolved Solids (TDS) and detailed component concentrations for each solution are provided in Table 2 to ensure reproducibility.

**Table 2:** Composition of synthetized seawater solutions.

No.	Solutions	NaCl (g/L)	Na_2_SO_4_ (g/L)	CaCl_2_ (g/L)	MgCl_2_.6H_2_O (g/L)	KCl (g/L)	Clay (g/L)	CTAB (g/L)	TDS (ppm)
1	SW	24.123	4.021	1.140	9.230	0.902	1.5	0.386	39,416
2	SW5d	12.061	2.010	0.570	4.615	0.451	1.5	0.386	19,707
3	SW5d.3Ca	12.061	2.010	3.420	4.615	0.451	1.5	0.386	22,557
4	SW5d.3Mg	12.061	2.010	0.570	27.690	0.451	1.5	0.386	42,782
5	SW5d.3SO4	12.061	12.063	0.570	4.615	0.451	1.5	0.386	29,760

#### Clay particle

Research indicates that clay minerals possessing high cation exchange capacity (CEC), particularly kaolinite, significantly influence low salinity water flooding processes through their interfacial interactions^13,16^. For this investigation, kaolinite was used as a common clay mineral in sandstone reservoirs to study fluid-rock interactions between clay particles, CTAB surfactant, ionic species, and heavy components of fresh oil. The experiments utilized high-purity kaolinite powder (average particle size: 0.85 μm). A comprehensive mineralogical characterization was performed by X-ray fluorescence spectroscopy (XRF), and quantitative elemental composition data are presented in Table 3. It is worth noting that the CEC and average particle size were 4.9 meq/100 g and 0.85 μm, respectively.

**Table 3:** The elemental composition of the clay particles characterized by XRF.

Compositions	Result (%mass)
SiO_2_	41.29
AL_2_O_3_	37.21
Fe_2_O_3_	0.5
CaO	0.37
TiO_2_	5.75
Na_2_O	0.65
K_2_O	0.53
Loss in Ignition (LOI)	11.89

### Method

#### Sample preparation

**Fig. 1:**
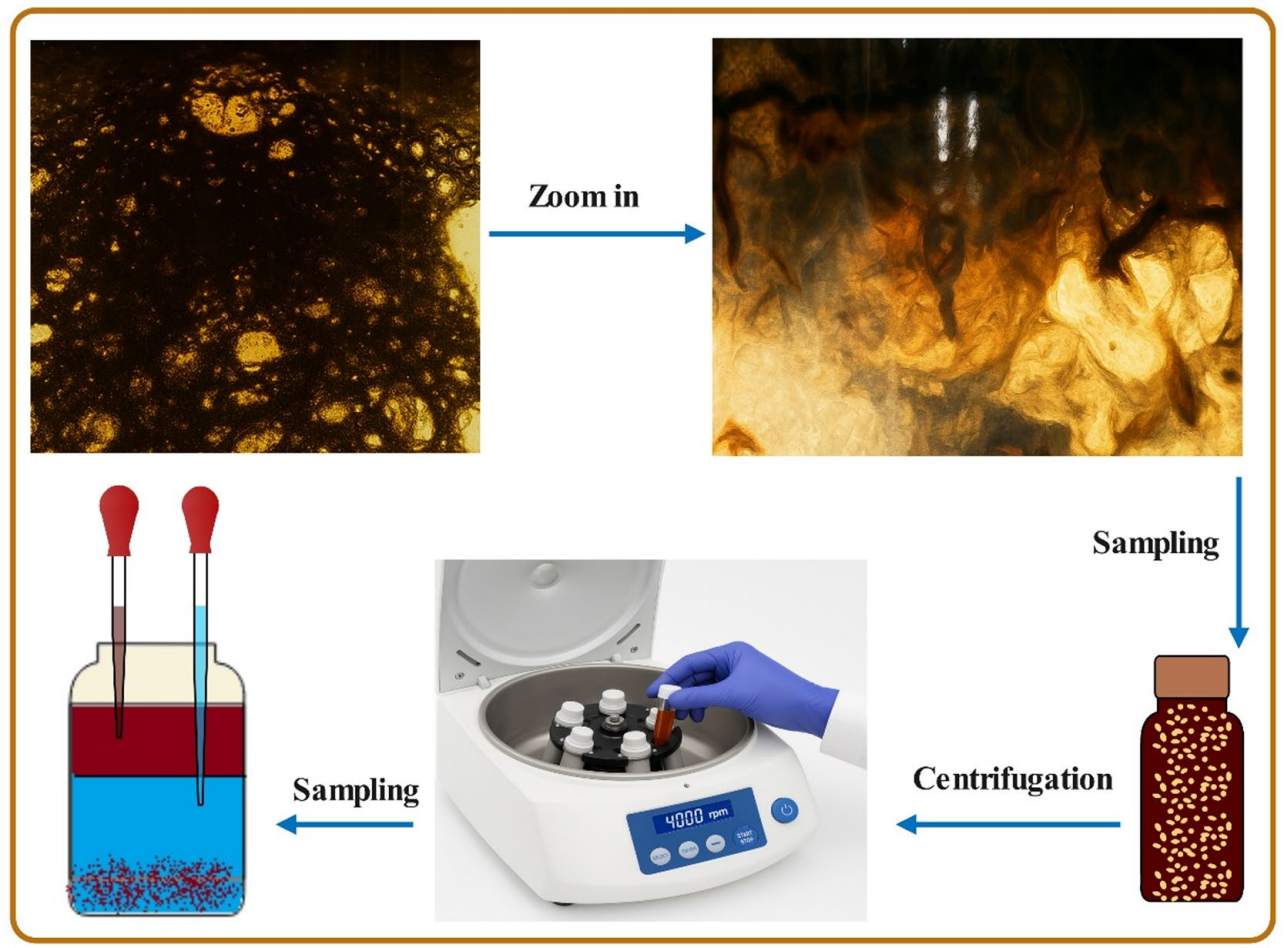
Schematic diagram illustrating the step-by-step methodology used to analyze emulsion stability in this study.

To prepare oil-water emulsions, static bottle tests were conducted. A 50 mL volume of synthesized water (containing CTAB and clay) and ion-tuned water was combined with a ratio of 60:40 (brine: oil) volume^46^. The mixture was homogenized using a magnetic stirrer for 6 h, followed by sonication in an ultrasonic bath (580 W, 37 kHz) for 20 min to ensure uniform dispersion. The prepared emulsions were then aged in an oven at 90 °C for 20 days. After aging, the emulsion samples were transferred into centrifuge tubes and subjected to centrifugation at 4000 rpm. Phase separation was monitored at 4-minute intervals until complete separation was achieved^16,46,47^. Emulsion stability was assessed based on the amount of oil, water, and solid fractions obtained after centrifugation, as can be seen in Fig. 1.

For subsequent experiments, the oil phase was carefully extracted from the top layer of the centrifuged samples. To ensure sample purity, the collected oil underwent double centrifugation to remove any residual water, clay particles, or dissolved gases. The final purified oil samples, referred to as “aged oil-synthesized seawater,” were stored for further analysis. All experimental procedures were performed in triplicate to ensure methodological reproducibility and verify data accuracy.

#### Interfacial tension

The interfacial tension between the aged oil and distilled water was measured to evaluate changes in the oil’s polar functional group concentration^22^. Since IFT is influenced by ion type and salinity, distilled water was used to investigate the effect of oil polarity modifications.

The pendant drop method was employed for IFT determination. In this technique, oil was injected through a needle into a transparent cell filled with distilled water^22,48,49^. The experimental setup consisted of a high-resolution camera, a computer for image analysis, a cubical glass chamber, a precision syringe pump, and fluid delivery lines. After allowing the oil-water system to stabilize, the shape of the pendant drop was captured and analyzed using specialized software to calculate the IFT.

#### Zeta (ζ) potential

The zeta potential of both aged and fresh oil samples was analyzed using a MICROTAC Wave particle size analyzer to assess the surface charge characteristics of oil droplets suspended in distilled water. All measurements were conducted at 25 °C and atmospheric pressure to ensure consistency. To verify reproducibility, each test was conducted five times, and the average value was reported.

##### Sample preparation procedure

The following steps were followed for sample preparation and treatment^10^:

Suspension Preparation: A 1 wt% oil-in-water suspension was prepared by mixing aged oils with distilled water.

Homogenization: The suspensions were sonicated for 1 h to ensure uniform dispersion of oil droplets.

Aging Process: The prepared samples were placed in the oven at 90 °C for 3 days to simulate thermal aging effects.

Measurement Conditions: The zeta potential was measured at room temperature (25 °C) to evaluate the stability and surface charge of the oil droplets.

#### Asphaltene extraction

The IP-143 standard test was employed to quantify the asphaltene content in aged oil samples. The procedure was carried out as follows^50^:

1. A mixture of aged oil and n-heptane (1:20, g/mL) was prepared and subjected to reflux condensation for 1 h to ensure complete asphaltene precipitation.
2. The solution was then transferred to a cool, dark, and dry environment and left undisturbed for 24 h to facilitate asphaltene aggregation.
3. The precipitated asphaltenes were filtered using Whatman 42 filter paper (0.7 μm pore size).
4. The filter paper containing asphaltenes was placed in a Soxhlet extractor, where it underwent reflux washing with n-heptane to remove residual maltenes.
5. Subsequently, the asphaltenes were further purified by refluxing with toluene to dissolve and extract them from the filter paper.
6. The toluene-asphaltene solution was collected and placed under a fume hood to allow for complete toluene evaporation.

#### ATR (Attenuated total reflection)-FTIR (Fourier transform infrared spectroscopy)

To characterize the functional groups of asphaltene extracted from aged oils, ATR-FTIR spectroscopy was performed using a PerkinElmer Frontier spectrometer. The analysis focused on identifying key functional groups, including sulfoxide (S = O), as well as other polar and aromatic structures^10,22,51^. In this regard, a homogeneous sample was prepared by mixing asphaltene powder with potassium bromide (KBr) at a 1:20 (w/w) ratio. The mixture was then compressed under high pressure to form transparent pellets suitable for ATR-FTIR absorbance measurements. Finally, the ATR-FTIR spectra were recorded in absorbance mode across a wavenumber range of 400–4000 cm⁻¹. The integral area of transmission peaks (from baseline to baseline) was calculated to quantify functional group contributions. It is worth mentioning that Origin software was used for spectral deconvolution and numerical integration of peak areas.

#### Asphaltene onset point precipitation

The asphaltene onset point (AOP) was determined by monitoring changes in oil viscosity upon incremental addition of n-heptane (a precipitating agent). The procedure was conducted as follows^22^:

1. A fixed volume of aged oil was titrated with varying volume percentages of n-heptane (vol oil: n-heptane vol) at 25 °C.
2. Each mixture was homogenized at 400 rpm for 1 min to ensure uniform dispersion.
3. The viscosity of the oil-heptane mixture was measured after each addition (as per the method outlined in Sect. 2.2.7).

The AOP was characterized as the critical heptane concentration where a gradual decrease in viscosity (due to dilution) was followed by a sudden increase. This indicated asphaltene aggregation and precipitation.

#### Viscosity measurement

The dynamic viscosity of fresh oil and emulsion-separated oil phases was determined using a Rotational Viscometer Rheolab-QC (Anton Paar, Germany) equipped with a CC27 spindle, at a constant temperature of 25 °C and a constant shear rate of 100 s⁻¹. Each measurement was performed 4 times to ensure repeatability, with the mean value reported.

## Result and discussion

To find a proper connection between the important compounds of fresh oil such as asphaltene and the variation of properties of water-oil interface and the oil bulk, several parameters related to the fresh oil before and after 20 days were evaluated in the following sections.

### Interfacial tension (IFT)

**Fig. 2:**
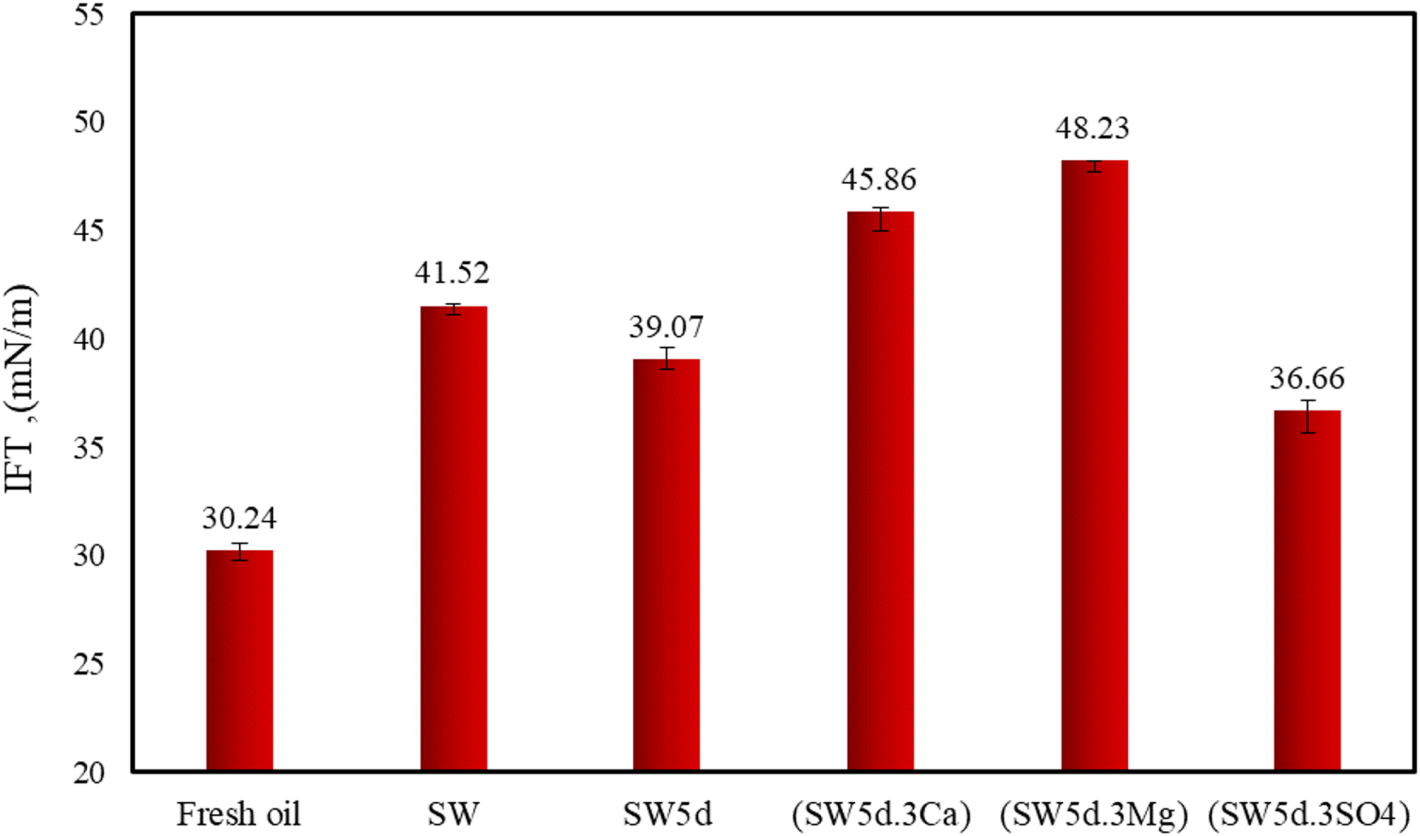
Comparison results of interfacial tension in fresh and aged oil samples.

This section investigated IFT variations between fresh and aged oil (extracted from emulsion phases). Previous studies demonstrate that IFT values are highly sensitive to multiple factors, including surfactant presence, pH, temperature, pressure, polar fraction concentration (particularly asphaltenes and resins), and interfacial salinity^33,52^. The interfacial affinity of asphaltenes and resins is recognized as a critical determinant of IFT modification. In our experimental research, aged oil phases were subjected to IFT measurements in distilled water under standardized conditions (25 °C, 1 atm), maintaining consistent aqueous phase properties while varying only the oil composition. This approach enabled investigation of oil polarity changes due to exposure to the ion-tuned seawater with CTAB and clay particles. Figure 2. presents the equilibrium IFT values for all oil samples.

Figure 2 demonstrates that the IFT value between distilled water and aged oil phases varied significantly depending on the ionic composition of the contacting aqueous phase in the presence of clay and CTAB. Quantitative analysis revealed that aged oil samples exhibited IFT values ranging from 36.66 to 48.23 mN/m, with specific values depending on accompanying ion types and salinity. Notably, sulfate-enriched solutions consistently yield lower IFT values compared to other ionic compositions, suggesting a higher concentration of polar components in oil bulk.

For example, the IFT value for aged oil-(SW5d.3Mg) and aged oil-(SW5d.3SO4) was observed to be 48.23 mN/m and 36.66 mN/m, respectively. The observed IFT differences among oil samples exposed to various smart water solutions indicate that oil chemistry was affected by aqueous phase interactions. This phenomenon primarily results from the migration of amphiphilic hydrocarbons from the bulk oil phase to the emulsion interface, consequently modifying the oil-water interfacial and oil phases’ properties. This reduction in IFT value of oil exposed to sulfate-dominated systems indicated a lower tendency of asphaltene to migrate toward the interface compared to cation-enriched seawater.

As illustrated in Fig. 2, the IFT of oil contacted with SW (41.52 mN/m) was higher than in diluted seawater (SW5d) (39.07 mN/m). It should be noted that increasing the concentration of divalent cations (Mg²⁺ and Ca²⁺) in seawater further elevated IFT compared to both SW and SW5d. Reduced seawater salinity diminishes the availability of active cations, which are critical for interactions with clay minerals and the mobilization of polar oil components (e.g., asphaltenes and resins)^5,22^. The following mechanisms may contribute to the phenomena observed in the emulsion phase:

1. Cationic Bridging between Clay, Divalent Ions, and Polar Oil Constituents: Divalent cations such as Ca²⁺ and Mg²⁺ can simultaneously coordinate with negatively charged sites on clay surfaces and polar functional groups (e.g., carboxylic, phenolic, and pyrrolic moieties) in asphaltenes^10,22,53^. This “asphaltene–ion–clay” bridging promotes the aggregation of oil-wet particles at the oil–water interface, stabilizing water-in-oil emulsions. Similar bridging effects have been documented in colloidal systems and crude oil–brine–mineral interfaces, showing that Ca²⁺ often exhibits stronger bridging capacity than Mg²⁺^10^.
2. Asphaltene-ion Complexation: Multivalent ions can form coordination complexes with heteroatom-containing groups in asphaltenes, altering their solubility and aggregation behavior. This interaction can either stabilize or destabilize asphaltene aggregates depending on ion type and concentration. Studies on heavy oil systems have shown that sulfate (SO₄²⁻) can disrupt these complexes through competitive adsorption, mobilizing less-polar components to the interface, whereas cation-rich environments tend to promote aggregation and precipitation^22,46^.
3. Electric Double Layer (EDL) Expansion upon Salinity Reduction: Lowering brine salinity increases the thickness of the EDL around both clay particles and asphaltene aggregates, enhancing electrostatic repulsion between dispersed particles^37,54^. This can weaken inter-particle attractions and destabilize aggregates. Previous smart-water flooding studies have reported that EDL expansion is a key mechanism for asphaltenes migration^37,46^.
4. Synergistic Effect of CTAB and Clay Particles: Cationic surfactant molecules (CTA⁺) from CTAB interact electrostatically with negatively charged silanol (Si–OH) groups on clay surfaces, forming a monolayer of CTA⁺ cations^46,55^. This interaction leads to the formation of oil-CTA+-clay complexes, reducing the hydrophilicity of clay particles and enhancing their tendency to partition into the oil phase^46^. This surface modification can alter interfacial properties, as observed in other CTAB–mineral systems, affecting emulsion stability.

In SW5d, the lower ionic strength reduced cationic bridging and complexation between ion, oil components, and clay particle, thereby decreasing the formation of asphaltene-ion-clay aggregates. Conversely, in SW, the high divalent cation concentration enhanced these interactions, promoting greater migration of polar components to the interface. Additionally, the reduced salinity in SW5d expanded the EDL, creating more space for polar component migration. However, in the presence of CTAB molecules, the EDL thickness could be partially occupied by surfactant molecules, favoring the migration of less-polar oil components and further suppressing asphaltene-ion and asphaltene-ion-clay interactions. Consequently, aged oil-SW exhibited lower concentrations of asphaltenes and resins compared to aged oil-SW5d, corroborating the IFT trends observed in Fig. 2.

The presence of active divalent cations (Ca²⁺ and Mg²⁺) migrated less significantly toward the interface and mainly remained in the aged oil phase, as the result of the IFT trend showed in Fig. 2. It is important to note that interfacial tension measurements revealed a difference between calcium-enriched seawater (SW5d.3Ca) and magnesium-enriched seawater (SW5d.3Mg): the IFT of aged oil-(SW5d.3Ca) (~ 45.86 mN/m) was lower than aged oil-(SW5d.3Mg) (48.23 mN/m).

This disparity could be attributed to the lower charge density of Ca²⁺ compared to Mg²⁺, which facilitated the preferential accumulation of CTAB at the oil-(SW5d.3Ca) interface. Calcium ions tend to form more stable complexes with surfactants like CTAB than magnesium ions. This suggested a competitive interfacial adsorption process between calcium ions and CTAB molecules, where the superior surface activity of CTAB significantly reduced clay and calcium’s interfacial participation^4,5,55^. Thus, the polar component could not significantly migrate toward the interface and mainly remained in the aged oil phase.

On the other hand, the higher charge density of Mg²⁺ had the ability to promote greater adsorption of polar components (such as asphaltenes and resins) in aged oil-(SW5d.3Mg) in the presence of clay particles. Consequently, the dominant interfacial mechanism shifts toward the formation of asphaltene-ion and asphaltene-ion-clay complexes, leading to a more pronounced alteration in IFT relative to aged oil-SW, aged oil-SW5d, and aged oil-(SW5d.3Ca).

The aged oil-(SW5d.3SO4) sample demonstrated a significantly lower IFT value (9.2 mN/m) compared to the aged oil-(SW5d.3Ca) sample. This substantial reduction can be primarily attributed to the decreased concentration of active divalent cations at the oil–water interface in the sulfate-enriched system, which in turn enhanced CTAB accumulation and strengthened the formation of oil–CTA⁺–clay complexes^53,56^. The electrostatic repulsion between sulfate ions (SO₄²⁻) and the negatively charged polar components of fresh oil, such as asphaltenes and resins, likely disrupted their adsorption at the oil–water interface^10,57^. his disruption reduces the tendency of these high-molecular-weight, surface-active species to compete with CTAB molecules for interfacial occupancy.

Furthermore, in sulfate-rich brine, the negatively charged clay surfaces can show weakened interactions with asphaltenes, since both the clay particles and sulfate ions carry a net negative charge^10^. This electrostatic repulsion diminished asphaltene–clay complex formation, thereby reducing the role of clays as bridging agents in the system. As a result, sulfate ions can significantly weaken the synergistic interactions between asphaltenes, cations, and clay particles, effectively suppressing cationic bridging mechanisms and limiting the formation of asphaltene–ion–clay aggregates at the oil-(SW5d.3SO4) interface. This shift in interfacial composition can favor a CTAB-dominated interface, which is changed with the observed lower IFT values in sulfate-enriched sample compared to cation-rich samples.

Under these conditions, the migration of polar components to the interface in this sample was lower than oils exposed to the SW and cation-enriched solutions, as shown in Fig. 2. Consequently, the IFT of aged oil-(SW5d.3SO4) remained relatively close the fresh oil.

It is important to emphasize that the observed interfacial changes could result from the influence of asphaltene or resin molecules as polar components of fresh oil. However, interfacial tension measurements alone cannot delineate the individual contributions of these components to the oil’s interfacial behavior. To address this limitation, Asphaltene Onset Point (AOP) measurement can serve as a complementary technique, providing critical insights into how asphaltene or resin fractions influence oil and emulsion properties under different aqueous-phase conditions.

### Asphaltene onset point precipitation (AOP)

**Fig. 3:**
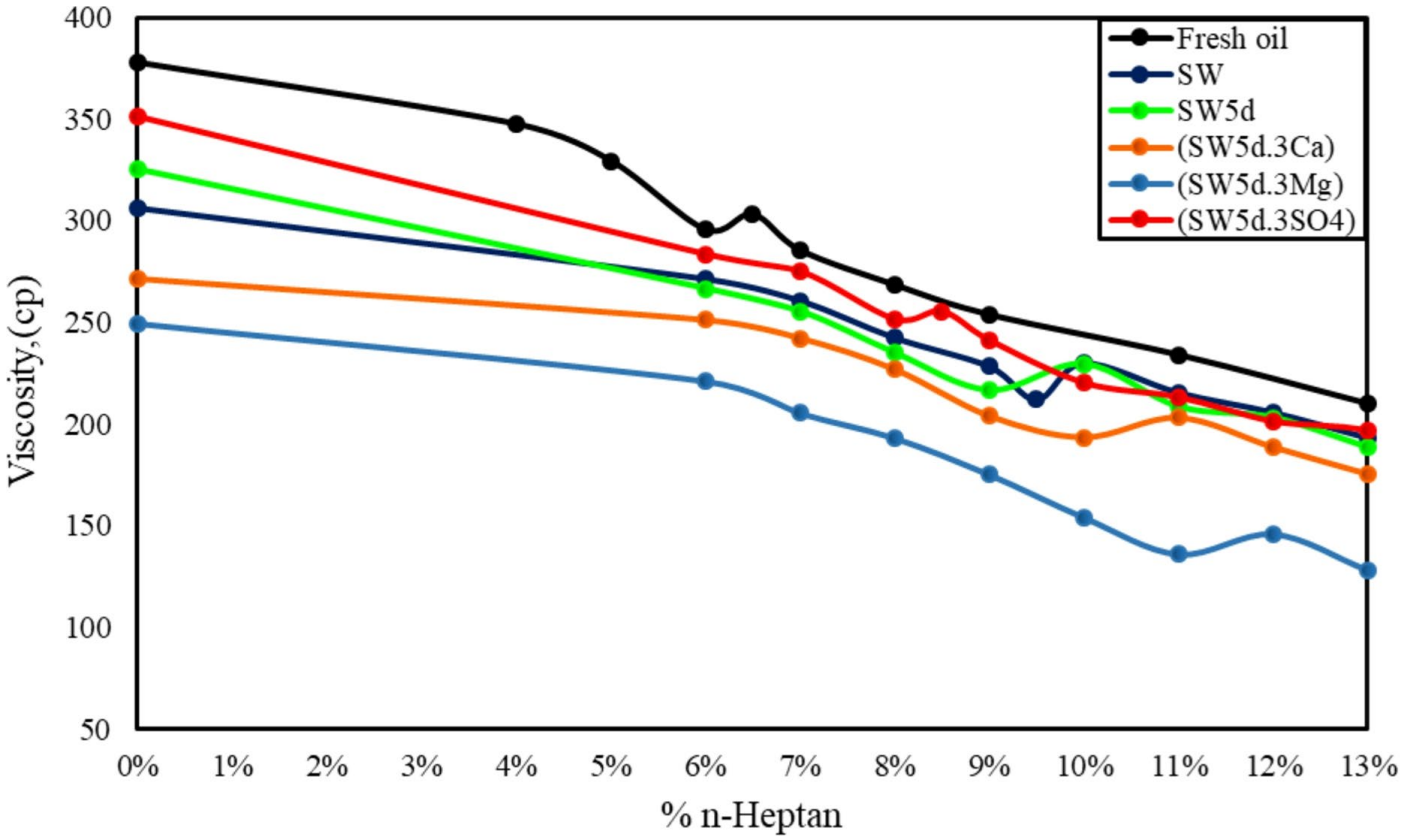
Onset point of asphaltene precipitation in oils exposed to smart water with clay and CTAB.

**Fig. 4:**
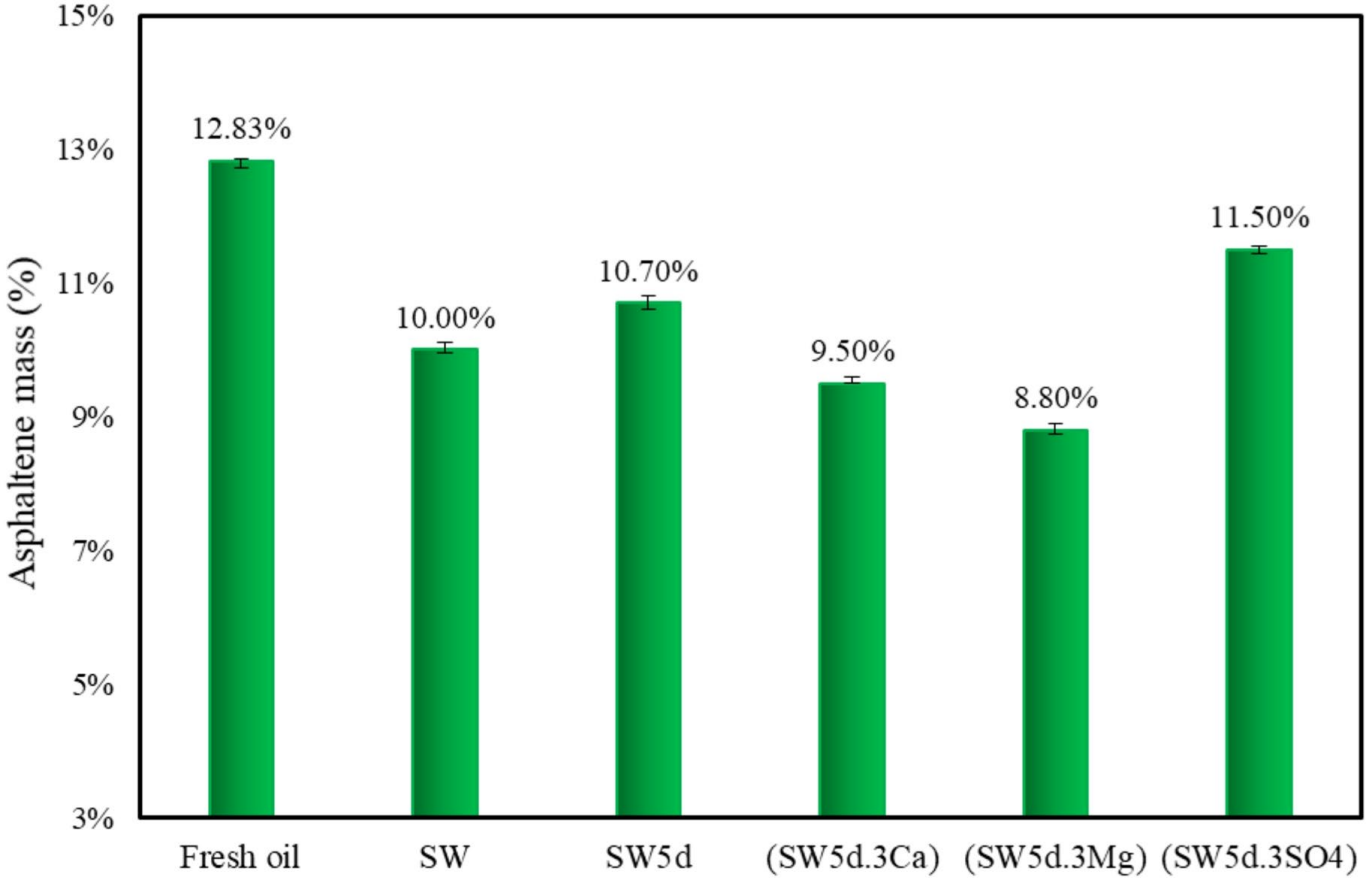
Asphaltene precipitation behavior under smart water flooding with CTAB and clay.

In this section, we conducted AOP measurements and quantified asphaltene concentrations for both fresh and aged oil samples. These analyses provided a comprehensive evaluation of the influence of asphaltene and resin molecules on emulsion behavior. The results are presented in Figs. 3 and 4.

As can be seen in Fig. 3, the observed increase in AOP (8–11%) across different aqueous phases stems from the distinct interfacial behaviors of asphaltenes and resins. As demonstrated by colloidal instability index (CII = 0.93), the fresh oil in this experimental study contained unstable asphaltenes that preferentially migrate to oil-water interfaces^48^. This interfacial accumulation led to oil phase depletion of the most unstable, high-molecular-weight asphaltene aggregates, leaving behind a more stable asphaltene population with higher precipitation resistance. Concurrently, resin molecules remain solubilized due to their higher hydrogen-to-carbon ratio^58,59^ and ability to form hydrogen bonds through polar functional groups^60^, resulting in a more homogeneous oil phase.

The AOP trends observed in Fig. 3. reveal distinct behaviors depending on the ionic composition of the aqueous phase. The AOP in aged oil-SW was approximately 9.5%, whereas in aged oil-SW5d, it was slightly lower at 9%, indicating a higher AOP in aged oil of seawater compared to diluted seawater. Furthermore, the reduction in asphaltene content was more pronounced in seawater (asphaltene mass = 10%) than in twice-diluted seawater (SW5d) (asphaltene mass = 10.7%), indicating enhanced asphaltene migration to the oil-water interface in high-salinity conditions, as illustrated in Fig. 4. This observation implies that asphaltenes exhibited greater interfacial activity than resins under these conditions.

The observed trends can be attributed to the influence of salinity on the migration behavior of CTAB and clay particles. At lower salinity (SW5d), the expanded EDL facilitated increased CTAB migration to the interface, competing with asphaltenes for active cations necessary for complex formation (asphaltene-ion and asphaltene-ion-clay). Due to CTAB’s high surface activity, its preferential adsorption at the interface reduced the available sites for asphaltene accumulation, increasing precipitation onset in aged oil-SW5d. Conversely, in seawater, the higher cation concentration and reduced EDL thickness limited CTAB’s interfacial presence. This enabled unstable asphaltenes to migrate more readily to the interface, and, in turn, delayed asphaltene precipitation^25,61^.

According to Fig. 3, the smallest change in AOP was observed in aged oil-(SW5d.3SO4) (8%), while oil exposed to magnesium- and calcium-modified brines (SW5d.3Mg and SW5d.3Ca) induced more significant AOP elevations (11% and 10%, respectively). This trend was corroborated by asphaltene content measurements (Fig. 4), which revealed the largest reduction in aged oil-(SW5d.3Mg) (asphaltene mass = 8.8%), followed by aged oil-(SW5d.3Ca) (asphaltene mass = 9.5%), with the least decrease in aged oil-(SW5d.3SO4) (asphaltene mass = 11.5%). Collectively, these findings suggested that asphaltenes can exhibit stronger interfacial affinity than resins, particularly in the presence of divalent cations and clay particles.

The suppressed AOP elevation in sulfate-rich systems (SW5d.3SO4) can be attributed to sulfate’s stabilizing effect on asphaltenes in the bulk oil phase. Unlike divalent cations, sulfate ions (SO₄²⁻) lack the ability to form stable interfacial complexes with asphaltenes^10^. It can be concluded that their migration reduced due to the accumulation of interface by CTAB molecules and oil-CTA^+^-clay complexes reduced. Furthermore, the pronounced interfacial activity of CTAB in sulfate-enriched systems likely competed with asphaltenes and clay for interfacial occupancy, further limiting their asphaltene-ion-clay interaction and precipitation onset^28^.

On the other hand, divalent cations (Ca²⁺ and Mg²⁺) could noticeably enhance asphaltene migration to the interface due to their high charge density, which facilitates the adsorption of asphaltene aggregates. This phenomenon was particularly evident in (SW5d.3Mg), which exhibited the highest AOP rise (11%). The superior efficacy of Mg²⁺ over Ca²⁺ can be explained by its smaller hydration radius and stronger electrostatic interactions, which promote tighter asphaltene-ion and asphaltene-ion-clay complexes and more efficient interfacial accumulation. It is worth noting that to better analyze the molecular structure of asphaltene and its interfacial interactions, ATR-FTIR spectroscopy was employed in the following section.

### ATR (Attenuated total reflection)-FTIR (Fourier transform infrared spectroscopy) of asphaltene

**Fig. 5:**
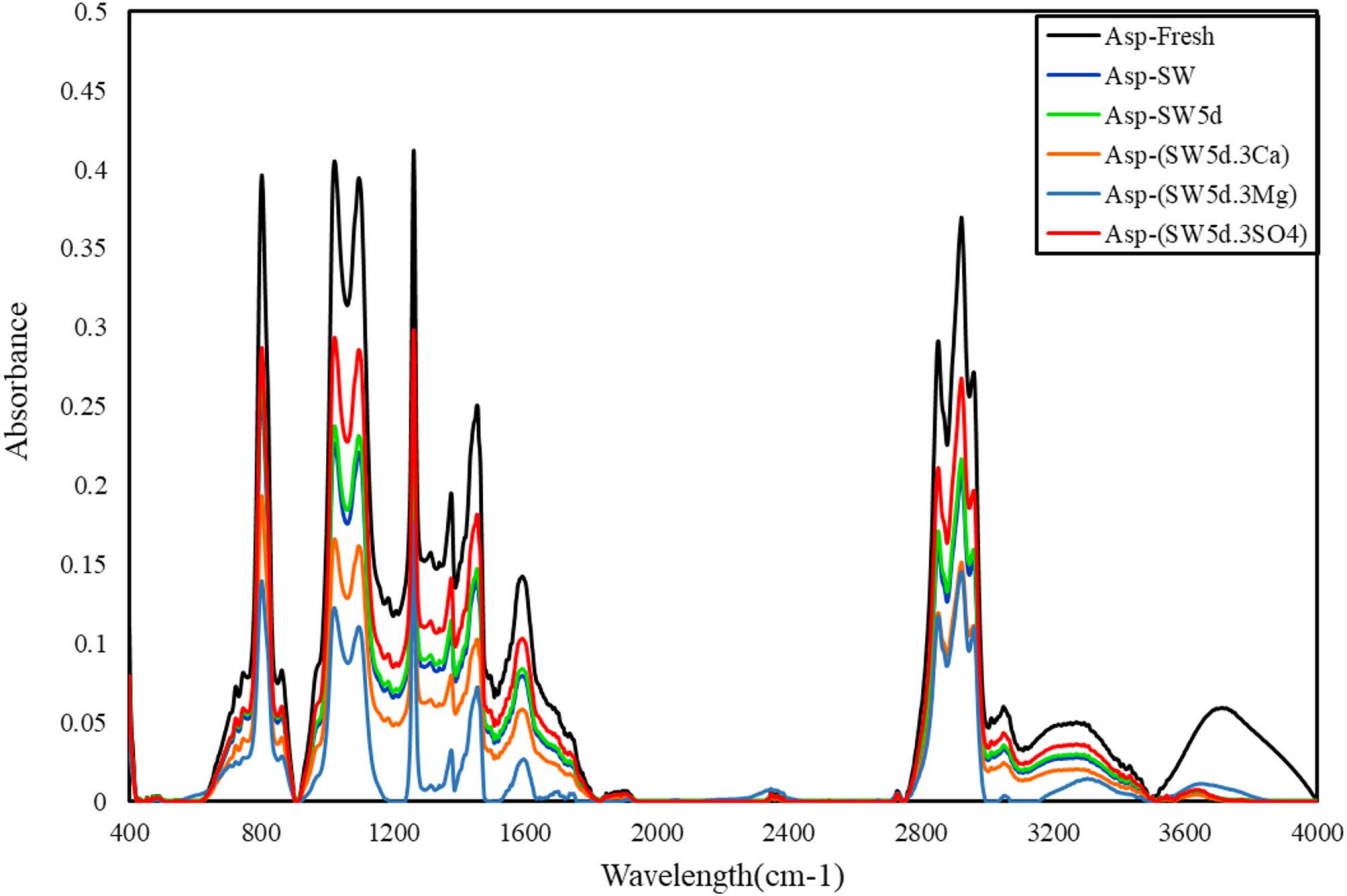
ATR-FTIR characterization of aged oils’ asphaltenes in presence of CTAB and clay.

As discussed in the AOP analysis, the observed variations in IFT, AOP values, and asphaltene concentration results were primarily influenced by the high-polarity functional groups present in the oil phase, particularly in asphaltene constituents. However, due to the complex molecular architecture of asphaltenes, which incorporates both high-polar and less-polar functional groups^53^, the precise mechanistic role of their chemical composition during adsorption at emulsion interfaces remains incompletely understood. To elucidate these phenomena, we employed ATR-FTIR spectroscopy, a powerful technique for the simultaneous detection of high-polar and less-polar functional groups^22,43^. This approach enabled detailed characterization of asphaltenes extracted from oil phases that had been exposed to ion-modified brines, in the presence of CTAB and clay particle. Figure 5. reports the comparative ATR-FTIR spectra of asphaltene fractions separated from aged oil samples.

As evidenced in Fig. 5., the ATR-FTIR spectra of asphaltenes extracted from the oil phase (Asp-brines) showed identical characteristic peaks to those extracted asphaltenes from original fresh oil (Asp-fresh). This spectral correspondence confirms the preservation of all functional groups throughout the emulsification process, with no observable elimination of existing peaks or formation of new vibrational modes. However, quantitative analysis reveals notable variations in peak intensities (measured from valley to valley), suggesting changes in the relative abundance of these functional groups. Table 4. provides a comprehensive assignment of the observed vibrational bands, including their corresponding functional groups and potential molecular associations.

**Table 4:** ATR-FTIR-identified functional groups and bond types in asphaltenes.

	Oil
Wavelength(cm^−1^)	Functional groups
3300–3700	O–H stretch (carboxylic acid or Alcohol)
3000–3150	C–H stretch in aromatic
2950–3000	CH3 asymmetric
2900–2950	CH2 symmetric (Alkane)
2800–2870	CH2 asymmetric stretch (Alkane)
1550–1650	C = C stretch (in-ring) aromatic
1450–1500	C–H bend or scissoring
1350–1390	C–H rock, methyl (Alkane)
1100–1220	C–O stretch (Alcohol or Esters or carboxylic acid)
1000–1030	C–O stretch (Alcohol or Esters)
850–890	C–H “oop” (Aromatic)
770–830	C-H (aromatic)
680–770	C–H rock, methyl, seen only in long chain alkanes

Figure 5. and Table 4. illustrate a comparative ATR-FTIR absorbance analysis of Asp-fresh and Asp-(brines) samples. The spectra reveal five characteristic bands:

1. A doublet at 2730 and 2935 cm⁻¹ corresponding to HC = O (carboxylic acid) and C-H axial stretching vibrations, respectively^10,51,53,62^;
2. A distinct peak at 1455 cm⁻¹ attributed to CH3 group deformation;
3. The 1375 cm⁻¹ band representing asymmetric CH3 stretching;
4. Aromatic C-H bending vibrations at 725 cm⁻¹; and.
5. Aromatic C-H stretching modes at 3050 cm⁻¹.

The spectral profile shows remarkable consistency with previous findings by Mahdavi et al.^10,22^, confirming the structural integrity of oil components during aging.

Quantitative indices were introduced to enable better comparison and analysis of the peaks in Fig. 5. These indices, ALI (aliphatic), LC (long-chain), ARO (aromaticity), and CA (carbonyl-associated), are critical for systematically comparing ATR-FTIR spectra of asphaltenes extracted from both aged and fresh oils. These indices, which were calculated using Eq. 2 to 5, correspond to specific functional groups, providing a standardized analytical framework^10,22,51^. Here, “A” denotes the integrated peak area of relevant bonds in the ATR-FTIR spectra. The obtained results are reported in Table 5.

2 $$\:ALI=\frac{{A}_{1460}+{A}_{1376}}{{A}_{1600}+{A}_{1460}+{A}_{1376}+{A}_{1030}+{A}_{864}+{A}_{814}+{A}_{724}+{A}_{743}+{A}_{2953}+{A}_{2923}+{A}_{2862}}$$

3 $$\:LC\:index=\frac{{A}_{724}}{{A}_{1460}+{A}_{1376}}$$

4 $$\:CA\:index=\frac{{A}_{1700}+{A}_{1650}}{{A}_{1700}+{{A}_{1650}+A}_{1600}}$$

5 $$\:ARO\:index=\frac{{A}_{1600}}{{A}_{743}+{A}_{724}+{A}_{814}}$$

**Table 5:** Quantitative value of various indices (ALI, A_S=O_, CA, ARO, LC).

no	Solutions	ALI	As = o	CA	ARO	LC
1	Asp-fresh	0.391	0.799	0.605	0.639	0.353
2	Asp-SW	0.289	0.612	0.501	0.442	0.284
3	Asp-SW5d	0.311	0.673	0.534	0.485	0.256
4	Asp-(SW5d.3Ca)	0.263	0.582	0.460	0.427	0.307
5	Asp-(SW5d.3Mg)	0.227	0.533	0.429	0.390	0.339
6	Asp-(SW5d.3SO4)	0.342	0.726	0.568	0.533	0.204

As illustrated in Eq. 2, the prominent peaks in the LC index correspond to less-polar functional groups within the asphaltene molecular structure. Table 5. represents that the LC index varies significantly across the samples, with Asp-(SW5d.3SO4) showing the lowest value (LC = 0.204), indicating a higher proportion of polar groups. On the other hand, Asp-(SW5d.3Mg) has the highest value (LC = 0.339), suggesting limited migration of less-polar asphaltene molecules at the interface. Intermediate values were observed for Asp-SW (LC = 0.284), Asp-SW5d (LC = 0.256), and Asp-(SW5d.3Ca) (LC = 0.307). These findings were confirmed by the obtained result in IFT section, where aged oil-(SW5d.3SO4) displayed minimal IFT change. It can be deduced that limited migration of polar asphaltene fractions to the interface. Besides, IP-143 analysis indicated a slight decrease in asphaltene concentration alongside an increased asphaltene onset point precipitation. The higher LC index values highlighted that less-polar groups in molecular structure of asphaltenes, which highlighted stronger interactions with CTAB molecules, preferentially migrated to the interface, influencing interfacial behavior.

The CA index reflected the presence of carbonyl functional groups, while the ARO index is attributed to aromatic component rings in polar and heavy asphaltene molecules^22,46,63,64^. These structural characteristics, polarity (governed by CA) and aromaticity (determined by ARO), collectively influence asphaltene stability in the oil phase and their aggregation in the emulsion phase^51,53^. A decrease in peak area in aged oil samples suggests that the concentration of polar and unstable polyaromatic asphaltenes diminished in the oil phase.

Based on the data from Table 5. and Fig. 5, the most significant reduction in ARO and CA indices was observed for Asp-(SW5d.3Mg) (ARO = 0.429, CA = 0.390), indicating a substantial decrease in aromatic and carbonyl-containing asphaltenes. The relative decline in these indices followed the order: Asp-(SW5d.3SO4) (ARO = 0.533, CA = 0.568) > Asp-SW5d (ARO = 0.534, CA = 0.485) > Asp-SW (ARO = 0.501, CA = 0.442) > Asp-(SW5d.3Ca) (ARO = 0.460, CA = 0.427).

In Asp-SW sample, the ARO and CA values were higher than in Asp-SW5d, representing that reduced salinity enhanced the interfacial affinity of CTAB molecules. Given CTAB’s strong surface activity, its preferential adsorption at the interface diminished interactions with polar asphaltenes, while promoting the migration of less-polar asphaltenes.

Conversely, in Asp-(SW5d.3Mg) and Asp-(SW5d.3Ca), the high concentration of divalent cations (Mg²⁺, Ca²⁺) increased the interfacial accumulation of polar polyaromatic asphaltenes, forming asphaltene-ion complexes. This led to a depletion of polar asphaltene molecules in the bulk oil phase. Additionally, clay particles further reduced asphaltene concentration via cationic bridging, forming asphaltene-ion-clay complexes, a phenomenon more pronounced with Mg²⁺ due to its smaller ionic radius compared to Ca²⁺.

In sulfate-rich seawater (SW5d.3SO4), the presence of SO₄²⁻ diminished the interfacial affinity of clay particle and aromatic and polar asphaltenes to accumulate at the interface, as corroborated by IFT and AOP measurements. Instead, CTAB dominated the interface, preferentially interacting with less polar asphaltenes.

The Aliphatic Index (ALI) reflects the relative abundance of aliphatic functional groups in asphaltene molecular structure, and a decline in this index signifies a decrease in aliphatic content^22,51^. It should be noted that sulfoxides (S = O), which are polar sulfur-containing compounds in the molecular structure of asphaltenes, can attach to aliphatic branches. Hence, polarizing specific aliphatic groups of asphaltenes. Consequently, the observed decrease in both indices contributed to the preferential adsorption of asphaltenes with higher aliphatic content at the oil-water interface, driven primarily by the polarizing effect of the S = O functional group.

The distinct variations depending on brine composition were observed for Aliphatic Index (ALI) and sulfoxide (A_S=O_) values for asphaltenes extracted from aged oils, with ALI ranging from 0.227 to 0.342 and A_S=O_ from 0.533 to 0.726 (Table 5). The highest ALI (0.342) and A_S=O_ (0.726) values were observed for aged oil-(SW5d.3SO4), followed by aged oil-(SW5d.3Ca) (ALI = 0.263, A_S=O_ = 0.582) and aged oil-(SW5d.3Mg) (ALI = 0.227, A_S=O_ = 0.533). This progressive decline suggests that divalent cations (Mg²⁺, Ca²⁺) promote the migration of polar and high-molecular-weight asphaltenes to the interface more effectively than sulfate (SO₄²⁻)-enriched brine.

The reduced ALI and A_S=O_ values in Mg-rich systems can indicate preferential accumulation of aliphatic and sulfoxide-containing asphaltenes, likely due to their higher polarity and affinity for interfacial complexation.

The observed trends are attributed to competitive interfacial interactions between asphaltenes, ions, and clay particles. In sulfate-enriched brine, the lower ionic interaction strength allowed CTAB molecules to dominate the interface, retaining more aliphatic/sulfoxide groups in the bulk oil (higher ALI/A_S=O_) However, Mg²⁺ and Ca²⁺ facilitate asphaltene-ion-clay complexes via cationic bridging, preferentially extracting polar aliphatic and sulfoxide-rich asphaltenes (ALI/A_S=O_ depletion). The smaller ionic radius of Mg²⁺ enhances its charge density, strengthening complexation compared to Ca²⁺^65,66^. These findings were approved by interfacial tension results, where Mg²⁺ systems exhibited the lowest IFT due to extensive asphaltene migration.

### Oil viscosity

**Fig. 6:**
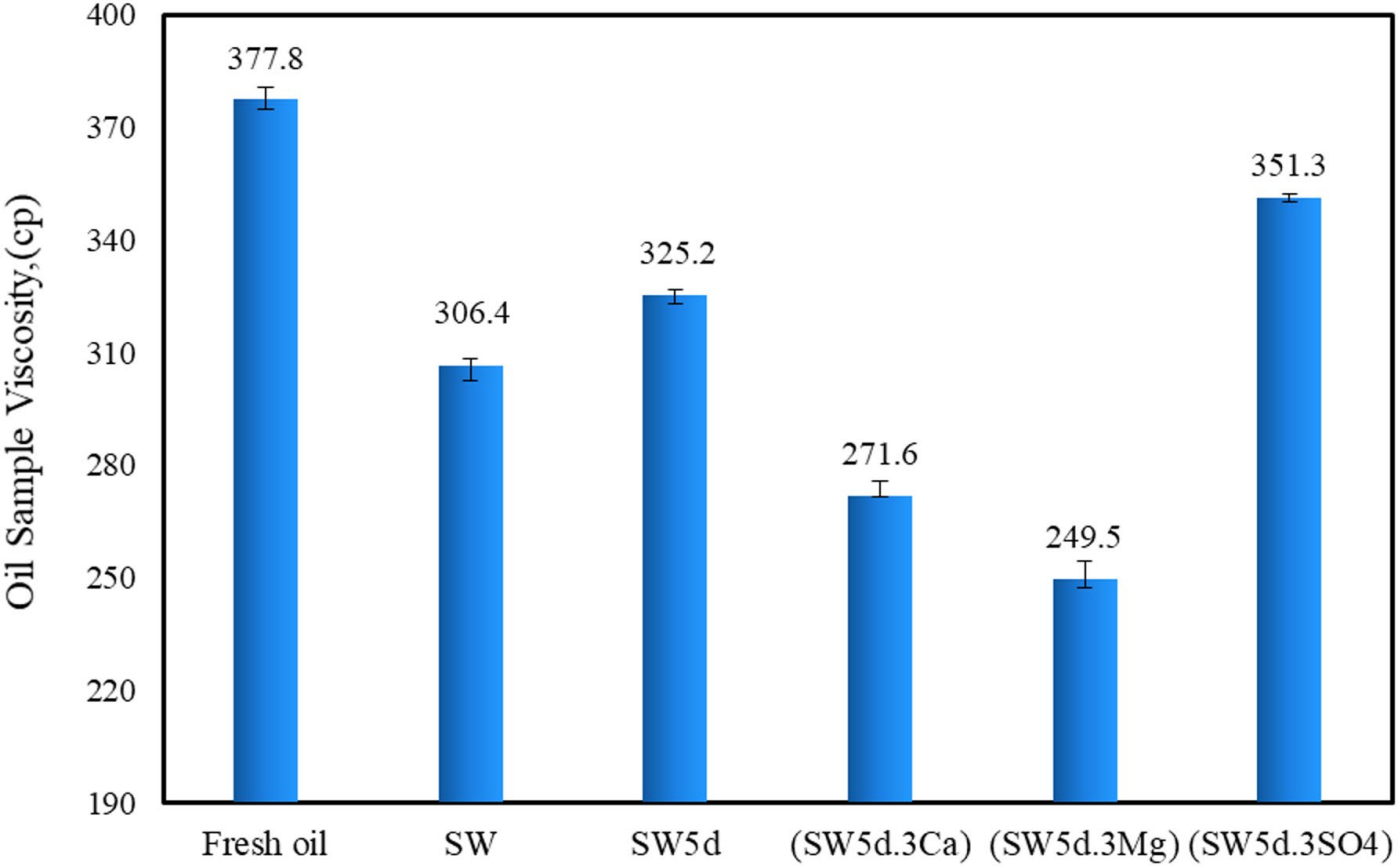
Viscosity measurements of oil after treatment with various smart waters containing CTAB and clay a constant shear rate of 100 s⁻¹.

Different studies have demonstrated that oil viscosity is intrinsically linked to both the concentration and molecular architecture of asphaltenes, a dominant component in heavy crude oils^10,28,67^. The complex interplay of asphaltene aggregation, polarity, and aromaticity significantly influences bulk viscosity properties, with higher asphaltene content typically correlating with elevated viscosity due to increased molecular interactions and reduced flow ability^68,69^. To clarify the role of asphaltene characteristics, this study evaluated the viscosity of both fresh oil and aged oil samples after exposure to aqueous phases. The obtained results are demonstrated in Fig. 6. While external factors like temperature and pressure are known to modulate viscosity, all experiments were conducted under isothermal (room temperature) and ambient pressure conditions to ensure observed changes related solely from compositional alterations, specifically, the redistribution of asphaltenes and other fractions at the oil-water interface.

Figure 6 demonstrates that the oil viscosity for aged oil-SW, aged oil-SW5d, aged oil-(SW5d.3Ca), aged oil-(SW5d.3Mg), and aged oil-(SW5d.3SO4), was measured roughly around 306.4 cP, 325.2 cP, 271.6 cP, 249.5 cP, and 351.3 cP, respectively.

As can be seen in Fig. 6, the viscosity of fresh oil decreased significantly after contact with seawater and divalent-ion-enriched brines (Mg²⁺, Ca²⁺), with the lowest viscosity observed for aged oil-(SW5d.3Mg). This trend correlates directly with the reduction in asphaltene concentration measured via the AOP analysis, where Mg²⁺ and Ca²⁺ brines improved the tendency of heavy, polar asphaltenes from the bulk oil phase to migrate toward the interface. The formation of asphaltene-ion complexes and cationic bridging (e.g., with clay particles) facilitated this extraction. Nonetheless, sulfate-rich brine (SW5d.3SO4) showed minimal viscosity reduction, as sulfate’s weaker interactions with asphaltenes left more aggregated networks intact.

Beyond concentration, the structural architecture of asphaltenes critically influences viscosity. Larger, more entangled branches—particularly those with heteroatoms (e.g., S, N, O)—enhance viscosity by promoting intermolecular interactions and physical entanglements. ATR-FTIR results revealed that these polar functional groups (e.g., sulfoxide S = O) are predominantly associated with aliphatic chains and peripheral aromatic rings, creating complex, cross-linked structures.

The data in Table 5. give a pronounced reduction in A_S=O_ and ALI indices for asphaltenes extracted from brine-contacted oils (Asp-brine) compared to fresh oil (Asp-fresh). For aged oil-(SW5d.3Mg), the lowest indices (A_S=O_ = 0.533; ALI = 0.227) coincided with the lowest viscosity (249.5 cP), while aged oil-(SW5d.3SO4) had the highest values (A_S=O_ = 0.726; ALI = 0.342) and viscosity (351.3 cP).

The results obtained from viscosity and IFT measurement show that CTAB molecules competed with cations (Mg²⁺, Ca²⁺) at the oil-water interface, influencing asphaltene migration and oil viscosity. In divalent cation-rich brines (SW5d.3Mg, SW5d.3Ca), the strong affinity of Mg²⁺ and Ca²⁺ for polar asphaltene groups (e.g., sulfoxides, S = O) promoted the formation of asphaltene-ion-clay complexes, particularly in the presence of clay particles. These complexes reduced entanglement of asphaltene molecular structure and developed the ability of migration of polar, high-molecular-weight asphaltenes from the aged oil bulk to the emulsion phase, reducing viscosity (e.g., 249.5 cP for SW5d.3Mg)^70,71^. On the other hand, sulfate-rich brine (SW5d.3SO4) lacks cationic bridging, allowing CTAB to dominate the interface. This retains polar asphaltene molecules in the oil, sustaining higher viscosity (351.3 cP).

Furthermore, the presence of clay in Mg²⁺/Ca²-enriched brines significantly enhances viscosity reduction by facilitating cationic bridging. Clay particles interacted with divalent cations (Mg²⁺ > Ca²⁺ due to smaller ionic radius) and polar asphaltene groups (S = O, aliphatics), forming ternary asphaltene-ion-clay complexes. This mechanism efficiently removed entangled asphaltene aggregates, disrupting the oil’s network structure. The result is a marked viscosity drop, as seen in (SW5d.3Mg) (lowest ALI/A_S=O_, lowest viscosity). In sulfate brine, the clay’s role diminished, leaving higher asphaltenes concentration in the aged oil phase.

As illustrated in Fig. 6, the observed viscosity reduction in aged oil-SW (306.4 cP) compared to aged oil-SW5d (325.2 cP) arises from distinct interfacial interactions governed by brine composition, asphaltene behavior, and surfactant dynamics. In SW5d, the moderate ionic strength enabled CTAB molecules to partially dominate the interface, interacting preferentially with less polar asphaltene fractions. This selective adsorption could leave some polar asphaltenes (e.g., sulfoxide-rich moieties) in the bulk oil but could reduce overall molecular entanglement, moderately lowering viscosity compared to aged oil-(SW5d.3SO4) and fresh oil.

Conversely, in SW, the competitive adsorption of polar asphaltenes, cations, and CTAB at the interface was enhanced. The high salinity water diminished CTAB’s effectiveness owing to their competitiveness with cations, allowing more polar asphaltene aggregates (particularly those with heteroatom branches) to migrate to the emulsion phase. The retained asphaltenes formed a less rigid and entanglement structure, decreasing viscosity relative to SW. Additionally, clay particles in SW could exhibit stronger flocculation due to a higher concentration of cations at the interface. Hence, further increases could occur in their efficiency in facilitating asphaltene-ion-clay complexation and viscosity reduction.

### Zeta potential

**Fig. 7:**
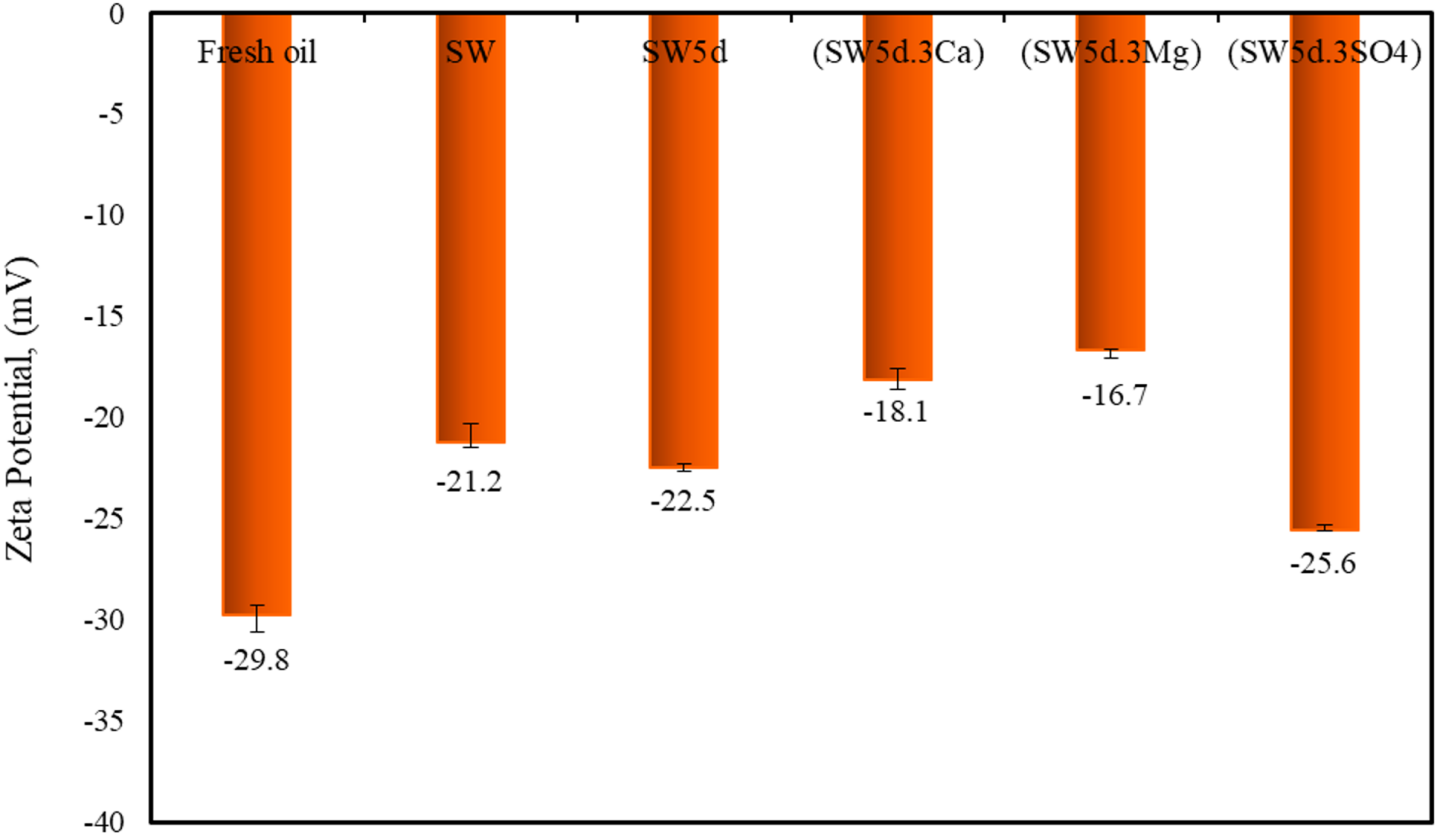
Zeta potential measurements of fresh and aged oil samples.

The zeta (ζ) potential serves as a critical indicator of colloidal stability, reflecting the surface charge of particles within the oil phase^72^. In fresh oil, the zeta potential can be notably influenced by the migration of asphaltenes, which carry polar and negatively charged functional groups (e.g., sulfoxides). Figure 7. reports the ζ-potential measurements of aged oils dispersed in distilled water. It is worth mentioning that the ζ potential of fresh oil was appeared at −29.8 mV.

As can be seen in Fig. 7, the ζ potential of examined aged oils was observed between − 16.7 and − 25.6 mV, which is lower than zeta potential of fresh oil. The zeta potential for aged oil-SW, aged oil-SW5d, aged oil-(SW5d.3Ca), aged oil-(SW5d.3Mg), and aged oil-(SW5d.3SO4) was − 21.2 mV, −22.5, mV −18.1 mV, −16.7 mV, and − 25.6 mV, respectively.

The sulfate-enriched system (aged oil-(SW5d.3SO4)) exhibited the most negative potential (−25.6 mV), closely resembling fresh oil’s charge characteristics, indicating minimal disruption to asphaltene’s polar groups. In contrast, calcium-containing brine (aged oil-(SW5d.3Mg)) showed the least negative potential (−16.7 mV), reflecting substantial charge neutralization. These trends arise from how different ions and presence of clay particles and CTAB molecules can modify the distribution and orientation of charged asphaltene groups at oil-water interfaces.

The presence of electronegative heteroatoms (e.g., sulfur, oxygen) within asphaltene molecular architectures confers significant surface-active properties, enabling these compounds to preferentially accumulate at oil-water interfaces^73^. As demonstrated by ATR-FTIR analysis, these polar functional groups engage in specific interactions with aqueous-phase cations through their anionic sites (e.g., sulfoxide), forming interfacial complexes that effectively decrease asphaltene concentration in the aged oil phase.

Figure 7 highlights that the sulfate-enriched system (SW5d.3SO4) has the most negative ζ-potential (−25.6 mV) among all tested conditions. This observation correlated strongly with the LC index data, which indicated preferential migration of less-polar asphaltene constituents to the interface in sulfate-rich environments. The presence of CTAB surfactant and clay particles further modulated this behavior through two competing mechanisms: (1) CTAB molecules dominate the interfacial layer owing to their superior surface activity in sulfate-enriched seawater solution, and (2) clay particles hindered asphaltene accumulation through weak electrostatic interactions. Consequently, the retention of charged asphaltene moieties in the bulk phase is enhanced, yielding higher absolute ζ-potential values^74,75^.

The observed ζ potential trends demonstrate distinct interfacial charge modifications governed by cation-asphaltene interactions. In standard and diluted seawater (SW/SW5d), the predominance of the presence of CTAB molecules rather than at the interface yielded moderately negative potentials (−21.2 to −22.5 mV). It should be noted that the reduction in seawater ionic strength (SW5d) significantly altered the interfacial equilibrium by decreasing cation availability, thereby enhancing the relative surface activity of CTAB molecules.

As demonstrated in Fig. 7, this ionic strength reduction directly correlates with a diminished propensity for asphaltene migration, as the reduced cation concentration (particularly divalent ions) limits the formation of asphaltene-ion and asphaltene-ion-clay complexes. The resultant interfacial environment becomes dominated by CTAB adsorption, which creates an electrostatic and steric barrier that further inhibits asphaltene migration. This phenomenon is quantitatively supported by the parallel decrease in both ζ-potential magnitude (−22.5 mV for SW5d versus − 21.2 mV for SW) and bulk phase asphaltene depletion (as evidenced by A_S=O_ index variations), confirming that lower ionic strength systems favor surfactant-controlled interfaces over ion-mediated asphaltene transport mechanisms.

Nevertheless, divalent cation-enriched brines (SW5d.3Ca/SW5d.3Mg) exhibit significantly reduced ζ-potential magnitudes (−16.7 to −18.1 mV) due to three synergistic mechanisms: (1) direct charge neutralization of asphaltene anionic groups (sulfoxides) through Ca²⁺/Mg²⁺ coordination, (2) cation-asphaltene interactions with aromatic cores, and (3) clay-facilitated cationic bridging^10,22^. The observed increase in zeta potential magnitude was more pronounced for aged oil-(SW5d.3Ca) than for aged oil-(SW5d.3Mg). This difference can be attributed to the stronger affinity of Ca²⁺ ions for forming stable coordination complexes with cationic surfactants such as CTAB. Calcium ions, owing to their lower hydration energy and larger ionic radius compared to Mg²⁺, can more readily displace water molecules from their hydration shell, facilitating direct electrostatic interactions with the positively charged head groups of CTAB^4,5,53^. This stronger complexation enhanced the adsorption of CTAB at the oil–water interface and improved interfacial packing density. Therefore, this influenced the distribution of polar oil components, including asphaltenes, and resulted in a greater change in surface charge characteristics compared to magnesium-enriched systems. These processes collectively diminished the polar asphaltene groups from the bulk phase, forming compact interfacial layers rich in aliphatic chains while depleting charged moieties. This phenomenon confirmed by parallel reductions in A_S=O_ indices and viscosity measurements.

### Emulsion phase

**Fig. 8:**
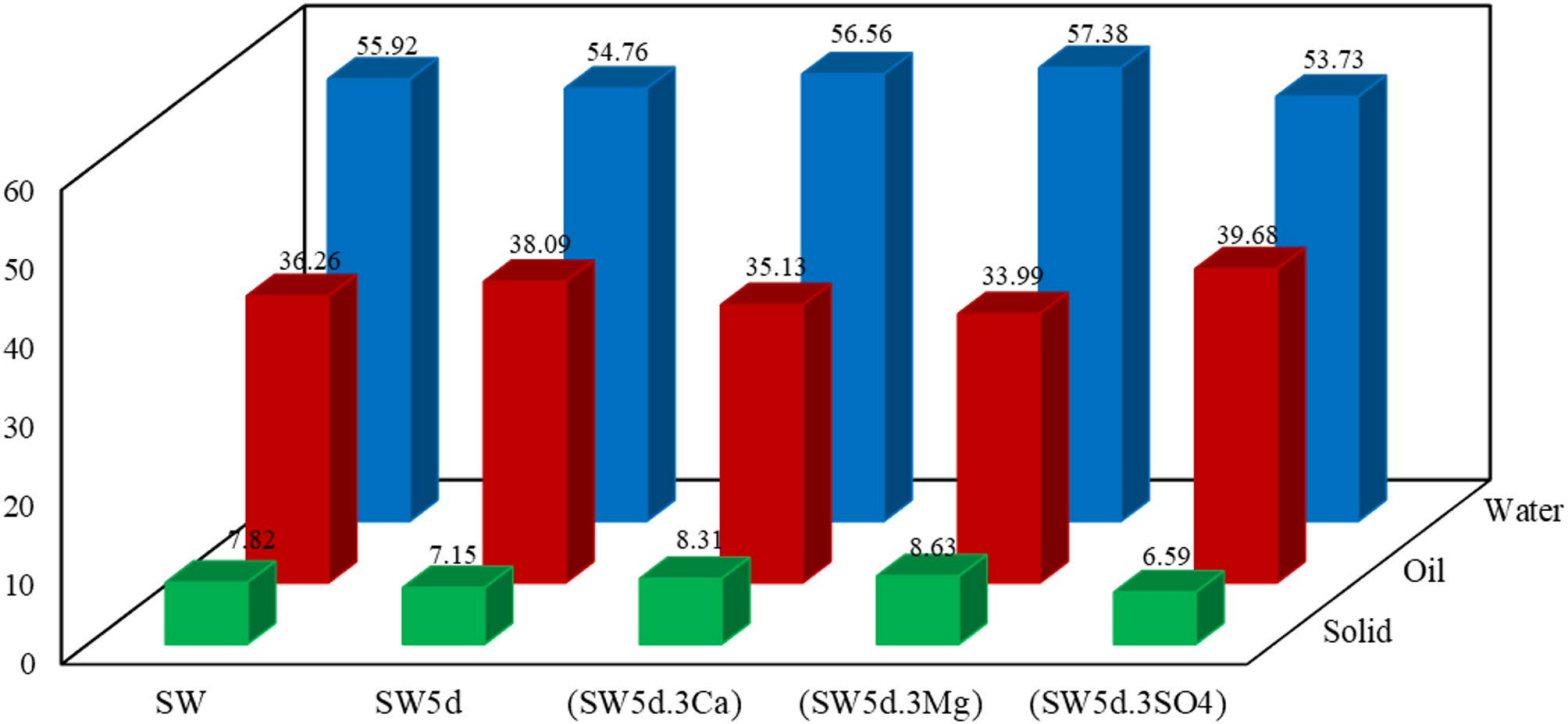
Phase-separated components following emulsion centrifugation.

The synergistic investigation of CTAB, ions, and clay can significantly influence the related phenomena of the fluid-fluid interface, including emulsion stability^76,77^. To investigate this effect, we evaluated emulsion stability by measuring the phase separation of water, oil, and solids in centrifuged samples. Quantitative analysis of the separated phases, as depicted in Fig. 8, provides key insights into emulsion stability across different experimental conditions.

As illustrated in Fig. 8, emulsion-(SW5d.3SO4) presented the highest oil phase separation from emulsion phase (oil = 39.68%), whereas emulsion-(SW5d.3Mg) showed the greatest solid phase extraction from emulsion sample (solid = 8.63%). Quantitative analysis revealed that emulsion-(SW5d.3Ca), emulsion-SW5d, emulsion-SW, and had oil separation values of 35.13%, 38.09%, and 36.26%, respectively, with corresponding solid depositions of 8.31%, 7.15%, and 7.82%.

Comparing seawater (SW) and diluted seawater (SW5d), emulsion-SW5d had higher oil separation (38.09%), while emulsion-SW produced more solids (7.82%), as indicated in Fig. 8. Dilution reduced cation competition, allowing greater CTAB adsorption at the interface, which promoted the migration of less polar oil components^78,79^. Conversely, the high cation concentration in seawater restricted CTAB mobility, leading to increased asphaltene and clay accumulation at the interface. This was further corroborated by lower interfacial tension and viscosity in aged oil-SW5d, confirming its superior emulsion stability. The findings align with previous studies demonstrating that reduced ionic strength enhanced surfactant efficiency^80,81^.

The enhanced oil separation in emulsion-(SW5d.3SO4) can be attributed to the reduced concentration of active cations at the interface, which facilitated greater adsorption of CTAB molecules due to their high surface activity and affinity for less-polar oil components. This process minimized the migration of asphaltenes and clay particles, as evidenced by the lower solid content, thereby improving emulsion stability. Previous studies have confirmed that such conditions favor oil recovery by reducing the formation of asphaltene-ion-clay and oil-CTA^+^-clay complexes^82^, aligning with the findings from zeta potential and viscosity analyses.

However, emulsion-(SW5d.3Mg) demonstrated the highest solid deposition and the lowest oil separation, indicating significant migration of asphaltenes and clay particles to the oil-water interface. Magnesium’s strong interaction with polar asphaltenes and clay via cationic bridging and complexation mechanisms led to greater asphaltene aggregation compared to calcium. Zeta potential and IFT measurements further supported this observation, showing that aged oil-(SW5d.3Mg) had lower asphaltene polarity and charge than aged oil-(SW5d.3Ca). Consequently, emulsion-(SW5d.3Mg) contained more asphaltene solids, whereas emulsion-(SW5d.3Ca) exhibited better oil interaction due to higher CTAB availability, enhancing trapped oil recovery^83^.

## Conclusions

This experimental study evaluated the effect of the molecular structure of asphaltene and its concentration under the simultaneous influence of low salinity water, CTAB, and clay, with a focus on emulsion stability and oil behavior. A novel sample preparation method was developed, followed by a series of tests to analyze oil phase and emulsion stability as a result of using the combination of smart water, CTAB, and clay particles. Based on the experimental findings, the following conclusions were drawn:

1. Sulfate-enriched brines yielded the lowest IFT (36.66 mN/m) due to suppressed asphaltene migration and enhanced CTAB adsorption, while divalent cations (Mg²⁺, Ca²⁺) elevated IFT (up to 48.23 mN/m) by promoting polar component migration via cationic bridging and complexation. Also, reduced salinity expanded the electric double layer, further modulating interfacial behavior.
2. Asphaltene precipitation onset (AOP) was governed by interfacial migration dynamics, influenced by ionic composition, salinity, and surfactant interactions. High-salinity and cation-enriched seawater conditions increased asphaltene migration to the interface, reducing bulk-phase instability. On the other hand, sulfate-rich seawater and diluted brine negligibly improved AOP by stabilizing asphaltenes and favoring CTAB adsorption.
3. The structural analysis of asphaltenes reveals that interfacial behavior was attributed to molecular polarity, aromaticity, and ionic interactions. Diluted seawater and sulfate-enriched seawater samples favored CTAB-dominated interfaces, retaining aliphatic/sulfoxide-rich asphaltenes in the bulk phase (higher ALI/AS = O), while divalent cations (Mg²⁺ > Ca²⁺) promoted interfacial accumulation of polar, aromatic asphaltenes via stronger cationic bridging, evidenced by lower ALI/AS = O and higher LC indices.
4. Divalent cations significantly reduced oil viscosity (aged oil-(SW5d.3Mg) and aged oil-(SW5d.3Ca)) by promoting the formation of asphaltene-ion-clay complexes, which extracted polar, high-molecular-weight asphaltenes from the bulk oil. The presence of clay further enhanced viscosity reduction in cation-rich systems by facilitating cationic bridging.
5. As indicated by zeta potential results, sulfate-enriched systems (SW5d.3SO4) strongly maintained negative potentials as a result of CTAB-dominated interfaces that limit polar asphaltene migration. In contrast, divalent cations (Mg²⁺/Ca²⁺) neutralized surface charge through three synergistic mechanisms: direct coordination with asphaltene anionic groups, cation-aromatic interactions, and clay-mediated bridging.
6. Sulfate ions and reduced salinity preferentially stabilized emulsions by surfactant enrichment at the interface, whereas divalent cations (Mg²⁺ > Ca²⁺) destabilized systems through polar component aggregation. The results provide a mechanistic framework for optimizing produced fluid treatment, improve trapped oil, and emulsion-breaking strategies in enhanced oil recovery operations.

## Data Availability

All data generated or analyzed during this study are included in this manuscript.
